# Treatment of Chorea

**Published:** 1861-01

**Authors:** 


*Treatment of Chorea.*—Dr. Stone, medical registrar of St. Thomas Hospital, has based the following conclusions upon fifty cases of chorea there treated during the year 1858: of sixteen cases treated with sulphate of zinc, thirteen went out cured, three relieved; but two of the latter were in a fair way of recovery, which may be set to the credit of the medicament. On the other hand, three of these ultimately cured, owed their improvement partly to ferruginous preparations. In one case the zinc had no effect whatever. The longest stay in the hospital among these sixteen cases was 123 days; shortest, 14; the average, 44.6 days.

Fourteen cases were treated during the same period with preparations of iron; all were cured. Longest time, 161 days; shortest, 6; average, 44.2 days.

Liquor potassæ arsenitis was employed in twenty cases; eigeteen cured, one relieved, one died. Longest stay, 55 days; shortest, 6; average, 26.3 days.

It remains a question whether the discrepancy beteen these results and those of some previous well-conducted observations is due to mere accident, or to some real difference in type between cases originating at different times and under dissimilar circumstances.—*Med. Times and Gazette.*

Dr. S. N. Pierce, of Cedar Falls, Iowa, has reported, in a former number of the *Boston Med. and Surg. Journal*, a case of chorea in a strong and plethoric girl of fifteen years, who was first treated with nitrate of silver and sulphate of zinc, without benefit. The addition of extract of stramonium seemed to control the disease somewhat, but soon that agent lost all its influence, even in incresased doses. Then the follownig pills were ordered, and conquered the convulsions: extracts of stramonium and hemlock, of each fifteen grains; strychnia, two grains; nitrate of silver, two scruples. Divide into thirty pills, three of which are to be taken during the day. The amount of strychnia and nitrate of silver was gradually increased.

